# Association of tumor mutation burden and epidermal growth factor receptor inhibitor history with survival in patients with metastatic stage III/IV non-small-cell lung cancer: A retrospective study

**DOI:** 10.6061/clinics/2021/e2251

**Published:** 2021-03-15

**Authors:** Yan Lan, Shuo Zhou, Weihong Feng, Ying Qiao, Xueming Du, Fenge Li

**Affiliations:** I Department of Oncology, Chifeng Songshan Hospital, Mongolia, China; II Department of Nuclear Medicine, Provincial Clinical Hospital of Fujian Medical University, Fuzhou, China; III Department of Oncology, Tianjin Beichen Hospital, Tianjin, China; IV Department of Melanoma, University of Texas M.D. Anderson Cancer Center, Houston, Texas, USA

**Keywords:** Lung Cancer, Hospice Care, EGFR Inhibitor, Overall Survival, Tumor Mutation

## Abstract

**OBJECTIVES:**

Lung cancer is the leading cause of cancer-related deaths worldwide. However, factors associated with the survival of patients with advanced non-small-cell lung cancer (NSCLC) who received only hospice care are largely unclear. In this study, we aimed to determine the prognostic factors correlated with survival in patients with advanced NSCLC who had undergone hospice care only.

**METHODS:**

A total of 102 patients with recurrent stage III/IV NSCLC after traditional treatment failure were investigated. Survival was measured from the date of enrollment to December 2019 or the time of death. Tumor tissues were collected, and DNA sequencing was performed to identify somatic mutations. Data on clinical factors of patients were collected and analyzed by univariate and multivariate analyses. Overall survival analysis was conducted using the Kaplan-Meier method.

**RESULTS:**

The 6-month, 1-year, and 2-year overall survival rates of the 102 patients with metastatic NSCLC were 17.65%, 3.92%, and 0.98%, respectively. The median overall survival of the 102 patients was 3.15 months. Tumor location in the peripheral lung, epidermal growth factor receptor (EGFR) inhibitor history, low tumor mutation load, adenocarcinoma, and poor performance status score were associated with prolonged survival compared with tumor location in the central lung, no EGFR inhibitor history, high tumor mutation load, squamous cell carcinoma, and good performance status score (*p*=0.045, *p*=0.003, *p*=0.045, *p*=0.021, and *p*=0.0003, respectively).

**CONCLUSIONS:**

EGFR inhibitor treatment history and tumor mutation load are risk factors for the overall survival of patients with stage III/IV NSCLC who have undergone only hospice care. These results provide a critical clinical basis for further study of nontraditional anti-tumor responses induced by EGFR inhibitors.

## INTRODUCTION

Lung cancer is the leading cause of cancer-related deaths worldwide (1). Non-small-cell lung cancer (NSCLC), which accounts for approximately 85% of all lung cancer cases, is often diagnosed at a late stage and has a poor prognosis (2). Traditional treatment strategies, including surgical resection and chemotherapy, are most commonly used in lung cancer treatment. However, the survival prognosis achieved with these conventional therapies is still unsatisfactory, with a 5-year survival rate of only approximately 15% (3-4). Recently, epidermal growth factor receptor-tyrosine kinase inhibitors (EGFR-TKIs) gefitinib, erlotinib, and AZD-92912 have been used to treat unresectable or recurrent lung cancer and have shown significantly improved clinical outcomes in patients with NSCLC with EGFR mutations (5-15). Nonetheless, tumors will inevitably develop drug resistance, and tumor recurrence has been observed (16-18).

Some patients who experienced disease progression, continued on conventional treatments following failures. It has been shown that continuing EGFR inhibitor treatment is beneficial in many patients even after they develop resistance to EGFR inhibitors on the basis of the hypothesis that a population of EGFR inhibitor-sensitive cells remains during disease progression, and resistant cells may be detected radiographically before widespread dissemination occurs (19). Other patients receive only hospice care. Patients who discontinue EGFR inhibitor treatment have a higher risk of symptomatic progression and increase in tumor size, which may lead to a much more rapid progression of the cancer (20-21). Despite the success of EGFR inhibitor treatment, questions on whether the benefits of continuing EGFR inhibitor treatment are temporary or long-term, how the overall survival is affected after EGFR inhibitor discontinuation, and what factors are correlated with overall survival in patients with metastatic stage III/IV NSCLC who receive hospice care remain unanswered. In this study, we aimed to determine the prognostic factors that are correlated with the survival of patients with advanced NSCLC who had received only hospice care.

## SUBJECTS AND METHODS

### Patient selection

**Figure 1:**
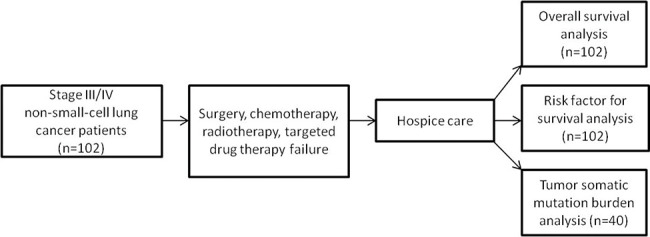
Flowchart of the study.

A total of 102 patients with stage III/IV advanced NSCLC between December 2015 and April 2019 were included in this study. The study protocol was in accordance with the ethical guidelines outlined in the Declaration of Helsinki, as revised in 2013, and was approved by the Tianjin Anti-Cancer Association and ethics committee of the Tianjin Beichen Hospital. Informed consent was obtained from all patients enrolled in this study. Patients were selected according to the following inclusion criteria: 1) stage III/IV NSCLC according to the National Comprehensive Cancer Network Clinical Practice Guidelines in Oncology (version 3.2016), Non-Small-Cell Lung Cancer Stage Classification; 2) disease progression on standard treatments, including surgery, chemotherapy, radiotherapy, and targeted drug therapy, and the absence of an active treatment option; 3) current hospice care only; and 4) a diagnosis of NSCLC confirmed by biopsy. Patients were excluded from the present study if they 1) did not meet the abovementioned criteria; 2) continued to receive antineoplastic therapies intended at prolonging survival, such as traditional cytotoxic, targeted, or immune-based therapies, despite disease progression; or 2) had incomplete medical records. It is noteworthy that patients with pleural effusion were all confirmed as having malignant pleural effusion by thoracentesis. None of the enrolled patients received any other survival-prolonging treatments during the follow-up period. All patient data were retrospectively collected from detailed hospital medical records after clinical sample detection. The clinical and demographic characteristics of all patients are summarized in Table 1. A study flowchart is shown in Figure 1.

### Hospice care

Hospice care is defined as supportive care at the end of life when life prolongation is not the primary treatment goal and disease-modifying therapies are no longer provided (22). Hospice care is particularly intended at improving the quality of life of patients through pain relief; treatment of fever and cough caused by lung tumor; and physical, psychosocial, and spiritual care. In this study, hospice care included the use of analgesics, antipyretic and cough medications, and antibiotics, with attention to psychological and spiritual aspects of care. None of the enrolled patients received antineoplastic treatments intended at improving survival, including conventional chemotherapy, radiotherapy, targeted drug therapy, or immunotherapy, during the follow-up period.

### Assessment of somatic tumor cell mutation by next-generation sequencing

We followed a genotyping panel designed to detect 508 cancer-associated genes on the basis of the biobanking system and in conjunction with the clinic and pathology laboratory (23). DNA was extracted from tumor samples of 40 patients immediately after biopsy using the TIANamp Genomic DNA Kit (TIANGEN) according to the manufacturer’s instructions. Then, exome libraries were constructed from the isolated DNA. Barcoded next-generation sequencing libraries were constructed (Hengjia Biotech), and exome captures were sequenced on the HiSeq X Ten System (Illumina, USA).

### Serological test for carcinoembryonic antigen (CEA), carcinoma antigen 125 (CA125), and carcinoma antigen 153 (CA153) and lymphocyte percentage analysis

Serum CEA, CA125, and CA153 levels were determined by a luminescence-based method using detection kits (Access CEA, Cat. #33200; Access OV Monitor, Cat. #386357; Access BR Monitor, Cat. #387620, Beckman Coulter Inc.) and subsequently measured using the Automatic Luminescence Immunoassay Analyzer (UniCel DxI 800, Beckman Coulter). Lymphocyte percentage was tested by fluorescence-activated cell sorting using the auto hematology analyzer (BC-6800, Mindray).

### Follow-up assessments

For survival analysis, all patients were followed up from the date of initial enrollment until December 2019 or until the time of death. Subsequent follow-up examinations included assessment of tumor biomarkers CEA, CA125, and CA153; peripheral blood T-cell counts; and tumor DNA sequencing of 40 patients from whom tissue samples were available.

### Statistical analysis

Univariate and multivariate analyses were performed to evaluate the possible factors that were correlated with patient prognosis and survival outcomes. Survival time was defined as the duration from the date of enrollment to the date of death or December 2019. The enrollment date for each patient in the study was the date of start of hospice care only. Survival curves were plotted, and overall survival rates were estimated using the Kaplan-Meier method and compared using the log-rank test or Cox’s proportional hazard model. Variables with *p*<0.6 on univariate analysis were entered into the multivariate model (Supplemental Table 1). The Cox regression method was used for multivariate analyses. Variables with *p*<0.05 were considered statistically significant. All statistical analyses were performed using the Statistical Package for Social Sciences (SPSS 16.0 for Windows; SPSS Inc., Chicago, IL) and GraphPad Prism 5.0 (USA).

## RESULTS

### Overall survival analysis of all patients with advanced stage III/IV NSCLC who had undergone hospice care only

**Figure 2:**
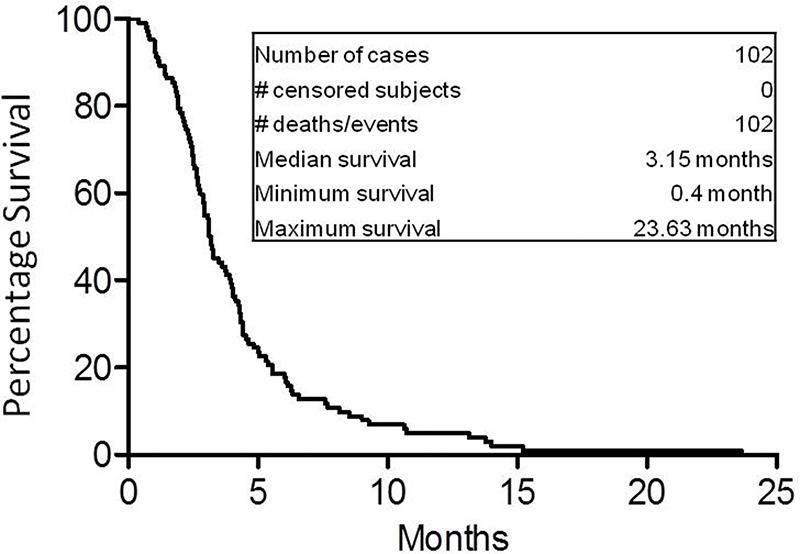
Overall survival (OS) curve of all 102 patients with advanced stage III/IV NSCLC. The 6-month, 1-year, and 2-year OS rates were 17.65%, 3.92%, and 0.98%, respectively. The median OS of all 102 studied patients was 3.15 months.

The 6-month, 1-year, and 2-year overall survival rates of the 102 patients with metastatic NSCLC were 17.65%, 3.92%, and 0.98%, respectively. The median overall survival of the 102 patients was 3.15 months (Figure 2), which was consistent with that in previous studies, which reported a median overall survival of 3-5 months (24-26). These results provide greatly important basic survival data to researchers evaluating new treatment strategies for patients with recurrent stage III/IV NSCLC who show progression on multiple conventional treatments.

### Correlation of EGFR inhibitor history with prolonged survival

**Figure 3:**
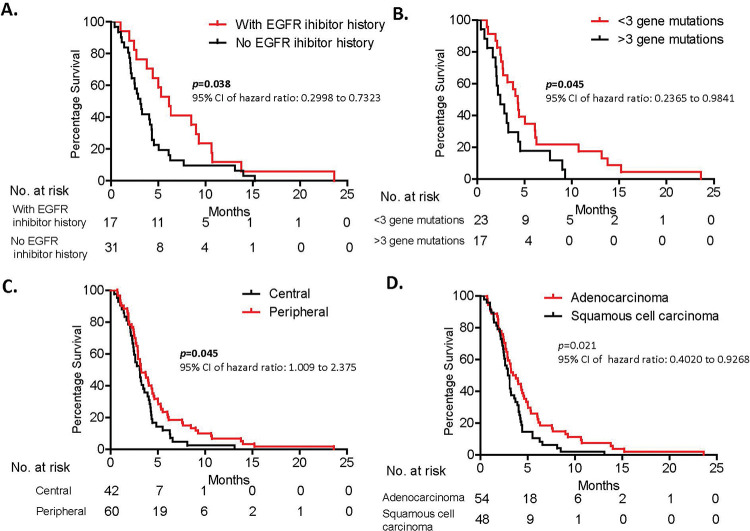
**A.** Patients with an EGFR inhibitor history showed better survival than did patients without an EGFR inhibitor history. **B.** Patients with a higher tumor mutation load survived for shorter periods than did patients with a lower tumor mutation burden. **C.** Patients with a tumor in the peripheral lung lived for significantly longer than did patients with a tumor in the central lung. **D.** There is statistical difference in overall survival between patients with squamous cell carcinoma and those with adenocarcinoma. Survival analysis was conducted using the Kaplan-Meier method.

Forty-eight of the 102 patients had undergone EGFR mutation evaluation, among whom 17 patients were found to have an EGFR mutation and EGFR inhibitor treatment history and 31 had no EGFR mutation or EGFR inhibitor treatment history. Detailed gene mutation signatures of each patient are shown in Supplemental Tables 2 and 3. The median overall survival time of patients with and without an EGFR inhibitor history were 6.13 months and 3.10 months, respectively (=0.038; 95% confidence interval [CI], 0.2998-0.9667) (Figure 3A, Table 2). This result suggests that EGFR inhibitor history was associated with a longer survival time than was no EGFR inhibitor history in patients. This leads to an extremely interesting question: Do EGFR inhibitors affect patient tumor progression and regression fate? Meanwhile, this result was based on a relatively small population of patients, and larger studies are needed to further confirm this finding.

### Higher somatic mutation load is associated with worse overall survival

We were able to collect tumor tissues from 40 of 102 patients and performed the 508 gene panel next-generation DNA sequencing analysis. A range of 0 to 9 mutations was detected in 40 patients (Supplemental Table 2). Univariate analysis of these 40 patients showed that >3 somatic mutations was associated with a shorter overall survival than with <3 somatic mutations (4.27 months *vs.* 2.43 months, *p*=0.045; 95% CI, 0.2365-0.9841) (Figure 3B, Table 2). This result implies that tumor cell mutation load impacts tumor cell growth, migration, and progression through possibly multiple unknown signaling pathways. Further mechanistic studies are necessary to better understand lung cancer progression.

### Potential correlation of demographic characteristics with patient prognoses

In addition to EGFR inhibitor history and tumor somatic mutation burden, we also found that tumor location within the lung and patient performance status score were associated with survival outcome. Patients with tumors in the central lung had extended survival compared with patients with tumors in the peripheral lung (3.25 months *vs.* 3.08 months, *p*=0.045; 95% CI, 1.009-2.375) (Figure 3C, Table 2). A patient performance status score-ECOG performance status of 0-2 was correlated with better survival than with an ECOG PS of 3 (4.58 months *vs.* 2.77 months, *p*=0.0003; 95% CI, 0.3125-0.7082 ) (Table 2). Furthermore, patients with adenocarcinoma and EGFR mutations showed a longer overall survival than did patients with squamous cell carcinoma and without EGFR mutation (*p*=0.021 and *p*=0.038, respectively). Further multivariate analysis showed that tumor pathology, ECOG performance status, and tumor site were independent factors associated with the overall survival of these patients (*p*=0.012, *p*=0.019, *p*=0.035, respectively). Curiously, clinical factors including pleural effusion; tumor size; tumor biomarkers CEA, CA125, and CA153; chemotherapy cycles; and brain metastases did not significantly affect survival in these patients and were therefore not considered risk factors (*p*=0.686, *p*=0.5509, *p*=0.219, *p*=0.809, *p*=0.445, *p*=0.344, and *p*=0.991, respectively; Figure 3D, Table 2). Our findings suggest that these clinical factors may not be correlated with survival in this terminal disease stage. However, these results are based on a small population of cases and particular background of patients and need to be further confirmed with extended studies of a larger size.

## DISCUSSION

Recently, much progress has been made toward advancing lung cancer treatments, such as EGFR mutation-based targeted therapies (27). However, the prognoses of late-stage lung cancer remain poor, as almost all patients eventually develop drug resistance and disease progression within one or two years (28-29). This is particularly important in Asia, as approximately 40% of patients with NSCLC carry EGFR mutations (23). What causes the difference between EGFR mutant and non-mutant tumors and how the tumor biology changes after EGFR inhibitor treatment failure remain largely unknown. Our group, along with many others, has previously shown that patients with EGFR-positive NSCLC have a worse overall survival compared with patients with EGFR-negative NSCLC (23,30-32). This result suggests that EGFR mutant tumor cells may be activated in multiple tumor proliferation and migration pathways that are associated with EGFR signaling. Therefore, blocking EGFR signaling has the potential to reverse or slow tumor progression.

Here, we report that patients with advanced stage III/IV NSCLC and an EGFR inhibitor treatment history have a significantly longer overall survival than do patients without an EGFR inhibitor treatment history (*p*=0.038). We have shown for the first time that EGFR inhibitor treatment may benefit even patients with NSCLC undergoing hospice care only, with a better overall survival. Another interpretation from our findings could be that patients with different EGFR statuses have distinct tumor biology. EGFR acts as a cytoplasmic kinase and mediates many/several important growth factors, signaling both extracellularly and intracellularly. We hypothesize that EGFR inhibitor treatment eliminates EGFR mutant cells while sparing non-mutant cells. Compared with EGFR mutant cells, these non-mutant cells proliferate at a much slower rate and, as a consequence, contribute to longer survival in patients previously treated with EGFR inhibitors. However, this result was based on a small sample size, and large-scale studies are needed to confirm our findings.

The correlation of survival and somatic mutation load has been reported in several different cancer types (33-35). High somatic mutation is correlated with decreased progression-free survival in multiple myeloma and tumor progression in melanoma (33-34). Interestingly, tumor mutation load is significantly correlated with the clinical outcome of anti-CTLA4 antibody and adoptive T-cell treatment in melanoma. The clinical benefit of anti-PD-1 treatment in lung cancer is also found to be strongly associated with tumor mutation load status (35). These results suggest that tumor cells with high mutation load are more aggressive in nature but are simultaneously more sensitive to immunotherapy. Consistent with these findings, we showed that patients with metastatic stage III/IV NSCLC and a higher tumor mutation load survived for shorter periods than did patients with metastatic stage III/IV NSCLC with a lower tumor mutation load (*p*=0.045). It is noteworthy that these patients were not under any antineoplastic treatment, and the result reflects the actual effect of tumor mutation load on overall survival.

Additionally, we analyzed other clinical factors that may be associated with the survival of patients with advanced stage III/IV NSCLC who were under hospice care only. Tumor site and performance status score were significantly correlated with patient survival, whereas tumor size; pleural effusion; tumor biomarkers CEA, CA125, and CA153; chemotherapy cycles; and brain metastases were not. Patients with tumors that initiated in or were localized to the peripheral lung lived longer than did those with tumors in the central lung. The reason for this is that tumors developing in the central lung are more likely to block the main airway and consequently lead to death. We also measured the overall survival of all patients, showing that the median overall survival was 3.15 months. The 6-month, 1-year, and 2-year overall survival rates were 17.65%, 3.92%, and 0.98%, respectively. These data establish a primary survival baseline for developing new treatment strategies in future studies.

Taken together, this study reveals that EGFR inhibitor history and tumor mutation load may influence the prognosis of patients even at the terminal disease stage. While the changes in tumor biology after EGFR inhibitor treatment and how immunogenic tumors continue to progress after treatment remain unknown, ongoing research is crucial to better understand the role of the EGFR signaling pathway and tumor mutation load in lung tumor cell fate determination and, in turn, help determine appropriate treatment modulation in patients with advanced lung cancer.

## AUTHOR CONTRIBUTIONS

Lan Y was responsible for the study design. Zhou S was responsible for the data collection and analysis. Feng W was responsible for the data collection. Qiao Y was responsible for the study design and project supervision. Du X was responsible for the study design and data analysis. Li F was responsible for the manuscript writing and data analysis.

## Figures and Tables

**Table 1:** Patients’ clinical and demographic characteristics at baseline.

Characteristic	Mean±SD or no. of cases (n=102)	Range
Sex (male-female)	57-45	-
Age (year)	65.64±11.20	28-91
Weight (kg)	62.50±9.45	40.5-83
Height (cm)	167.0±7.43	150-186
Smoking history (yes-no)	71-31	-
Pleural effusion (yes-no)	55-47	-
Tumor size (cm)	4.34±1.64	1.4-10.1
Tumor site (central-peripheral)	42-60	-
CEA (ng/mL)	71.40±88.73	0.91-1005
CA125 (U/mL)	110.03±257.67	11.47-2482.1
CA153 (U/mL)	50.10±103.37	4.5-1016.2
EGFR mutation (yes-no)	17-31**	-
*Previous treatments*		
Surgery (yes-no)	7-95	-
Local radiotherapy (yes-no)	89-13	-
Chemotherapy (yes-no)	73-29	-
EGFR inhibitor (yes-no)	17-85	-
Lymphocyte (%)	18.01±8.68	3.3-44.5
Brain metastases (yes-no)	11-91	-
Bone metastases (yes-no)	35-67	-
Tumor histology (SQ-AD)	48-54	-
ECOG PS (0/1/2-3)	31-71	-

** 48 of 102 performed EGFR mutation analysis.

**Table 2:** Factors associated with overall survival in 102 patients with advanced NSCLC according to univariate analysis.

Characteristic	Median or no. of cases	Median survival time (months)<median *vs.* ≥median(or left *vs.* right)	*p*-value	Chi-square	95% CI of hazard ratio
Sex (male-female)	57-45	3.07 *vs.* 4.23	**0.0305**	4.683	1.042 to 2.309
Age (year)	67	3.10 *vs.* 3.27	0.5676	0.3266	0.5887 to 1.337
Weight (kg)	162	3.27 *vs.* 3.10	0.6156	0.2520	0.6066 to 1.345
Height (cm)	170	3.97 *vs.* 2.92	0.1085	2.577	0.4759 to 1.077
Smoke history (yes-no)	71-31	3.2 0 *vs.* 3.10	0.7461	0.1048	0.6018 to 1.439
Pleural effusion (yes-no)	55-47	3.07 *vs.* 3.27	0.6862	0.1633	0.7309 to 1.610
Tumor size (cm)	4.35	2.93 *vs.* 3.23	0.5509	0.3557	0.5904 to 1.325
Tumor site (central-peripheral)	42-60	3.08 *vs.* 3.25	**0.0451**	4.013	1.009 to 2.375
CEA (ng/mL)	43.79	3.20 *vs.* 3.09	0.2187	1.513	0.5124 to 1.165
CA125 (u/mL)	65.99	2.85 *vs.* 3.95	0.8087	0.0586	0.7034 to 1.570
CA153 (u/mL)	34.39	3.47 *vs.* 3.02	0.4449	0.5835	0.5745 to 1.276
EGFR mutation (yes-no)	17-31*	6.13 *vs.* 3.10	**0.0381**	4.298	0.2998 to 0.9667
Number of tumor somatic mutations	3**	4.27 *vs.* 2.43	**0.0451**	4.017	0.2365 to 0.9841
*Previous treatments*					
Surgery (yes-no)	7-95	3.10 *vs.* 3.20	0.4324	0.6165	0.5879 to 3.459
Local radiotherapy (yes-no)	89-13	3.20 *vs.* 3.10	0.7088	0.1394	0.4807 to 1.646
Chemotherapy (yes-no)	73-29	3.20 *vs.* 2.93	0.5201	0.4137	0.7518 to 1.758
EGFR inhibitor (yes-no)	17-31*	6.13 *vs.* 3.10	**0.0381**	4.298	0.2998 to 0.9667
Chemotherapy cycles	3	3.60 *vs.* 3.10	0.3435	0.8973	0.5440 to 1.236
Lymphocyte (%)	19.6	2.87 *vs.* 4.01	0.0894	2.885	0.9484 to 2.097
Brain metastases (yes-no)	11-91	2.87 *vs.* 3.20	0.9912	0.0001	0.5281 to 1.880
Bone metastases (yes-no)	35-67	3.10 *vs.* 3.20	0.8146	0.0550	0.6287 to 1.440
Metastases (visceral-bone)	17-35	2.93 *vs.* 3.10	0.6422	0.3714	0.6540 to 2.243
Disease stage (III-IV)	38-64	3.20 *vs.* 3.10	0.6973	0.2791	0.7367 to 1.701
Tumor histology (SQ-AD)*	48-54	3.00 *vs.* 3.62	**0.0205**	5.367	0.4020 to 0.9268
ECOG PS (0/1/2-3)	31-71	4.58 *vs.* 2.77	**0.0003**	13.05	0.3125 to 0.7082

* 48 of 102 underwent EGFR mutation analysis;

** 40 of 102 patients underwent 508 gene panel analysis (23). SQ, squamous cell carcinoma; AD, adenocarcinoma

## APPENDIX

**Supplemental Table 1:** Multivariate analysis showed independent factors associated with the overall survival rates of 102 patients with NSCLC.

Variables	SE	*p*-value	Exp (β)	95% CI for Exp (β)
Age:>67 years *vs.* ≤67 years	0.570	0.710	0.809	0.265 to 2.472
Sex: female *vs.* male	0.865	0.446	1.933	0.355 to 10.528
Height: >170 cm *vs.* ≤170 cm	0.705	0.778	0.820	0.206 to 3.261
Tumor size: >4.35 cm *vs.* ≤5.35 cm	0.558	0.312	0.569	0.191 to 1.699
CEA: >43.79 ng/mL *vs.* ≤43.79 ng/mL	0.566	**0.030**	3.424	1.130 to 10.380
CA153: >34.39 u/mL *vs.* ≤34.39 u/mL	0.496	0.591	1.305	0.494 to 3.447
EGFR inhibitor history: yes *vs.* no	0.933	0.794	0.783	0.126 to 4.878
EGFR mutation: yes *vs.* no	0.546	0.051	2.897	0.994 to 8.441
Lymphocyte: >19.6% *vs.* ≤19.6%	0.495	0.908	1.059	0.401 to 2.796
Tumor pathology: SQ *vs.* AD*	0.793	**0.012**	7.275	1.536 to 34.445
ECOG PS: 0/1/2 *vs.* 3	0.652	**0.019**	4.626	1.289 to 16.602
Tumor site: central *vs.* peripheral	0.907	**0.035**	6.796	1.148 to 40.223
Chemotherapy cycles: ≥3 *vs.* <3	0.694	0.176	0.391	0.101 to 1.524
Chemotherapy: yes *vs.* no	0.797	0.066	4.327	0.908 to 20.628
Number of tumor somatic mutations: ≥3 *vs.* <3	0.724	0.077	3.591	0.870 to 14.829

* SQ, squamous cell carcinoma; AD, adenocarcinoma.

Bold values indicate significant difference.

**Supplemental Table 2:** Gene mutation results of 40 patients according to 508 gene panel analysis.

Patient ID	No. of gene mutation	Gene	Base mutation	Amino acids mutation
Pt. 12	1	TSC2	c.4519T>C	p.S1507P
Pt. 15	3	EGFR	c.2573T>G	p.L858R
		TP53	c.859G>T	p.E287*
		CDK4	c.71G>T	p.R24L
		EGFR	/	1.7copy number again
Pt. 19	9	TP53	c.337T>G	p.F113V
		RB1	c.1450_1451del	p.M484Vfs*8
		CD22	c.A139A>G	p.R47G
		PIK3C2A	c.1201C>T	p.R401C
		EPHA5	c.2767C>A	p.P923T
		NEK11	c.25A>G	p.K9E
		STAG2	c.3446A>T	p.H1149L
		EPHA5	c.688T>G	p.Y230D
		ATM	c.4966A>G	p.K1656E
Pt. 21	1	ERBB2	c.338G>A	p.R113Q
Pt. 22	1	EGFR	c.2573T>G	p.L858R
Pt. 23	6	RAD52	c.1037C>A	p.S346X
		CREBBP	c.1651C>A	p.L551I
		TEK	c.2029C>A	p.Q677K
		PIK3C3	c.718G>A	p.G240S
		MPL	c.1120A>G	p.T374A
		PIK3C2G	c.3299_3306dup	p.Y1103Vfs*15
Pt. 25	2	TP53	c.469G>T	p.V157F
		CDKN2A	c.250G>A	p.D84N
Pt. 26	7	NOTCH2	c.2548G>A	p.E850K
		APC	c.4666dup	p.T1556Nfs*3
		TP53	c.532del	p.H178Tfs*69
		MS4A1	c.161C>A	p.A54D
		KEAP1	c.1337A>T	p.E446V
		IGF1R	c.758A>G	p.H253R
		MLH3	c.2032A>G	p.N678D
Pt. 28	1	EGFR	c.2235_2249del	p.745_750del
Pt. 31	9	PTCH1	c.C3853>T	p.Q1285X
		KMT2D	c.10636C >T	p.Q3546X
		CARD11	c.3179G>A	p.C1060Y
		EXT1	c.1813C>T	p.R605W
		TRAF7	c.694C>T	p.R232W
		DNMT3A	c.1574C>T	p.A525V
		CCND3	c.391G>A	p.A131T
		ERBB2	c.1830G>T	p.K610N
		TAF1	c.3650G>A	p.R1217H
Pt. 35	2	CHUK	c.464T>C	p.V155A
		PTCH1	c.2225G>A	p.R742H
Pt. 39	1	TP53	c.527G>T	p.C176F
Pt. 42	2	KRAS	c.34G>A	p.G12S
		TP53	c.775G>T	p.D259Y
Pt. 44	9	ABL2	c.2948C>T	p.A983V
		BRCA2	c.8187G>T	p.K2729N
		DOCK2	c.4768A>G	p.R1590G
		ERBB2	c.380G>A	p.R127Q
Pt. 44	7	EGFR	c.2573T>G	p.L858R
		TP53	c.584T>C	p.I195T
		CDKN2A	c.315C>A	p.D105E
		NTRK1	c.2026C>T	p.R676C
		ALK	c.3311C>T	p.S1104F
		FGFR2	c.160C>T	p.P54S
		EGFR	/	1.7copy number again
Pt. 47	2	EGFR	c.2237_2254del	p.E746_S752delinsA
		EGFR	c.2255C>T	p.S752F
		BRCA1	c.718C>T	p.Q240X
Pt. 50	5	AKT1	c.607C>T	p.Q203X
		BRAF	c.1568C>T	p.P523L
		FGFR1	c.748C>T	p.R250W
		NF1	c.4998G>C	p.R1666S
		GOPC	c.1151G>A	p.R384H
Pt. 53	7	EGFR	c.2573T>G	p.L858R
		KMT2C	c.817delG	p.V273Wfs*82
		NFE2L3	c.1826_1829del	p.C611Tfs*22
		SMC1A	c.3103C>T	p.R1035X
		FAT3	c.12637C>T	p.R4213C
		SMAD4	c.608C>T	p.P203L
		PRKCG	c.614G>A	p.R205Q
Pt. 63	3	EGFR	c.2573T>G	p.L858R
		KRAS	c.35G>A	p.G12D
		TSC2	c.4594C>T	p.Q1532X
Pt. 64	2	STK11	c.1062C>G	p.F354L
		TP53	c.430C>T	p.Q144X
Pt. 65	6	NTRK1	c.865C>T	p.Q289X
		PMS2	c.80G>T	p.C27F
		ATM	c.7358G>C	p.R2453P
		SMAD4	c.608C>T	p.P203L
		TSC1	c.2696C>G	p.T899S
		PTEN	c.235G>A	p.A79T
Pt. 66	1	TP53	c.524G>A	p.R175H
Pt. 67	2	EGFR	c.2573T>G	p.L858R
		TP53	c.430C>T	p.Q144X
Pt. 68	4	DDR2	c.1267C>T	p.Q423X
		MET	c.1039G>A	p.A347T
		PTCH1	c.4144C>T	p.H1382Y
		HER2	c.2579C>T	p.A860V
Pt. 69	5	EGFR	c.2232_2233ins	p.I744_K745insKIP VAI
		MSH3	c.1180C>T	p.Q394X
		AR	c.1369_1377del	p.G457_G459del
		PTCH1	c.1628G>A	p.R543H
		TSC2	c.2023G>A	p.A675T
Pt. 70	2	PIK3CA	c.1633G>A	p.E545K
		TP53	c.524G>A	p.R175H
		TP53	c.1006G>T	p.E336X
Pt. 71	2	EGFR	c.2156G>C	p.G719A
		TP53	c.661G>T	p.E221X
		TP53	c.745A>T	p.R249W
Pt. 72	1	TP53	c.524G>A	p.R175H
Pt. 73	1	EGFR	c.2237_2251delinsT AG	p.E746_T751delins VA
Pt. 74	5	SMARCA4	c.1189C>T	p.R397X
		DPYD	c.1850C>T	p.T617M
		XPO1	c.740T>C	p.I247T
		PRKAA1	c.1382G>A	p.R461Q
		SMC1A	c.1990C>T	p.R664W
Pt. 75	2	EGFR	c.2235_2249del	p.745_750del
		TP53	c.724T>A	p.C242S
Pt. 76	5	U2AF1	c.101C>T	p.S34F
		CD22	c.2182C>T	p.Q728X
		SETD2	c.1814A>C	p.K605T
		TNFAIP3	c.1344G>C	p.W448C
		CARD11	c.157G>A	p.D53N
Pt. 77	3	MLH1	c.1192C>T	p.Q398X
		AR	c.2257C>T	p.R753X
		TSC2	c.5051_5068del	p.1684_1690del
Pt. 78	2	EGFR	c.2575G>A	p.A859T
		TP53	c.524G>A	p.R175H
Pt. 79	2	NF1	c.2023G>T	p.G675X
		TP53	c.532delC	p.H178fs
Pt. 80	2	EGFR	c.2573T>G	p.L858R
		EGFR	c.2240T>C	p.L747S
		TP53	c.832C>T	p.P278S
Pt. 81	3	ERBB2	Copy number gain	
		TP53	c.245_246insCACC	p.P82fs
		MET	c.3520G>T	p.V1174F
Pt. 82	0	None		
Pt. 83	2	TP53	c.536A>G	p.H179R
		TSC2	c.2023G>A	p.A675T
Pt. 84	2	U2AF1	c.101C>T	p.S34F
		TP53	c.994-1G>T	

**Supplemental Table 3:** EGFR mutation results of eight patients who had undergone single EGFR gene analysis.

Patient ID	EGFR status
Pt. 6	No EGFR mutation
Pt. 11	No EGFR mutation
Pt. 24	EGFR Exon21 p.L858R
Pt. 27	EGFR Exon 19 c.2235_2249del p.746_750del 7.17%
Pt. 41	No EGFR mutation
Pt. 48	No EGFR mutation
Pt. 60	EGFR Exon21 p.L858R
Pt. 34	No EGFR mutation
